# Cats show an unexpected pattern of response to human ostensive cues in a series of A-not-B error tests

**DOI:** 10.1007/s10071-020-01373-4

**Published:** 2020-03-29

**Authors:** Péter Pongrácz, Dóra L. Onofer

**Affiliations:** Department of Ethology, Institute of Biology, ELTE Eötvös Loránd University, Pázmány Péter sétány 1/c, Budapest, 1117 Hungary

**Keywords:** Companion cats, Ostensive signals, Perseveration, Domestication

## Abstract

It is an intriguing question whether cats’ social understanding capacity, including the sensitivity to ostensive signals (resulting in fast preferential learning of behavioural choices demonstrated by humans), would be comparable to that in dogs. In a series of A-not-B error tests, we investigated whether the ostensive or non-ostensive manner of human communication and the familiarity of the human demonstrator would affect the search error pattern in companion cats. Cats’ performance showed an almost completely different distribution of perseverative erring than earlier was shown in dogs and human infants. Cats demonstrated perseverative errors both during ostensive and non-ostensive cueing by the owner and also during non-ostensive cueing by the experimenter. However, unlike prior studies with dogs, they avoided perseverative errors during the experimenter ostensive cueing condition. We assume that the reliance on human ostensive signals may serve different purpose in companion dogs and cats—meanwhile in dogs, human ostension could support fast rule learning, in cats, it may have only a circumstantial attention-eliciting effect. Our results highlight the need of conducting further throughout experiments on the social cognition of cats, based on their own right beside the traditional cat–dog comparative approach.

**Electronic supplementary material:**

The online version of this article (10.1007/s10071-020-01373-4) contains supplementary material, which is available to authorized users.

## Introduction

According to the ‘natural pedagogy’ theory (Csibra and Gergely 2009), by paying dedicated attention to those actions of an adult that are accompanied by ostensive verbal cueing, human infants can rapidly learn such behavioural patterns (i.e. ‘rules’, search strategies) that otherwise would require elaborate explanation—obviously beyond the cognitive reach of a 10–12-month old infant. It has been found that companion dogs behave very similarly to toddlers in a Piagetian two-way search task—as individuals of both species showed strong preference for the location that was previously enhanced by ostensive signals by the demonstrator—thus committing the so-called A-not-B (perseverative) search error, when eventually the correct location for searching was different from the previously rewarded one (Topál et al. 2008, 2009). This was the first time when researchers connected perseveration in dogs with their responsiveness to ostensive cues, because other studies mostly investigated whether spatial and reward-related cues could influence dogs’ perseverative errors in tasks with consecutive hiding events (e.g. Gagnon and Doré 1992—with inconclusive evidence for perseveration; Péter et al. 2016—with solid evidence for perseveration). This behavioural analogy was further analysed, and Topál et al. (2009) found that meanwhile human toddlers can generalize between the ostensive–behavioural actions of multiple adult actors (thus signifying the role of ostensive communication in the transmission of culturally shared knowledge), dogs connect the relevance of ostensive–referential actions more rigidly to particular actors—therefore, in dogs, the specific sensitivity to human ostensive cues may function ultimately as the catalyst for rapid learning of human-given imperatives (Topál et al. 2009). As it was highlighted by others (Pongrácz et al. 2004; Téglás et al. 2012), the specific sensitivity to human ostensive signals in dogs may have an adaptive attention-getting function that elicits more effective interspecific communication and social learning.

The evolution of human-analogous socio-cognitive skills through domestication has strong empirical support from the past 2 decades of scientific investigations of dog behaviour (e.g., Kubinyi et al. 2007; Kaminski and Marshall-Pescini 2014). However, the role of domestication-related evolutionary events behind dogs’ responsiveness to human ostensive cues could be further strengthened by studying other domesticated species. The domestic cat offers a suitable, although still seldom investigated subject for this approach.

While the original function of the domesticated cat (namely, a semi-independent pest controller) in the anthropogenic niche remained mainly unchanged till present times (Hu et al. 2014; Themb’alilahlwa et al. 2017), and unlike dogs, cats retain a significant potential for forming feralised populations characterized by predatory lifestyle and the avoidance of humans (Daniels and Bekoff 1989), their role as a companion is becoming more dominant in industrialized societies worldwide. Cats are kept as pets in larger numbers in Europe than dogs (Zentek 2004; Downes et al. 2013), and according to various surveys, owners consider the cognitive capacities of their cats and their emotional bond with them comparable to the corresponding values with dogs (Vitulli 2006; Pongrácz and Szapu 2018). Although cats have not been clearly selected for different work tasks, as in the case of dogs (Coppinger and Schneider 1995; Gácsi et al., 2009), the need for a smooth coexistence with humans as companions could be a strong enough selective pressure on some of the socio-cognitive capacities of cats to become analogous to the ones seen in dogs. Indeed, researchers found that cats show similar performance to dogs in various tasks, for example in two-way object choice tasks, they follow proximal and distal pointing signals (Miklósi et al. 2005; human gazing, Pongrácz et al. 2019); they rely on their owner’s reaction when encountering unfamiliar objects (Merola et al. 2015); and they can recognize their owners’ voice (Saito and Shinozuka 2013; Takagi et al. 2019); as well as their own name (Saito et al. 2019). However, thus far, the only paper where the effect of human ostensive signals on cats was tested, showed a minimal effect only—the performance of cats in a gaze-following test was not affected by the presence or absence of ostensive signals, only the speed of establishing eye contact with the subject was enhanced by the ostensive cues (Pongrácz et al. 2019).

To our best knowledge, perseverative behaviour of cats has been seldom investigated apart from some older research reports in comparative psychology (Warren 1959; Warren and Kimball 1959). In those papers, Warren and colleagues reported for example that “stimulus perseveration seriously retards the solution of positional as well as object discriminations by cats”, where cats made fewer erroneous choices when they had only to rely on positional cues, meanwhile additional object-related cues deteriorated their performance. Furthermore, cats were also found having difficulty in overcoming negative transfer effects in discrimination problems. So far, the possible connection between perseveration and ostensive human signals has never been investigated in this species–although one could argue that companion cats would represent the perfect control model both for the ‘domestication’ and the ‘social environment’ hypotheses explaining the extraordinary socio-cognitive capacities of dogs. As the comparative studies run parallel on socialized wolves and dogs (Topál et al. 2009) could be considered as testing for evolutionary homologies (or the lack of them) in regard to dogs’ behaviour, the testing of companion cats would be suitable to show the generalizable effects of being adapted to the companion status.

In this paper, we tested companion cats (*N* = 38) in a two-way Piagetian object hiding paradigm developed by Péter et al. (2015), originally for testing perseverative responses in companion dogs and 2-year-old children. Briefly, a human demonstrator performed five consecutive, visible hidings of a food bait behind one of two non-transparent screens. The order of hiding events was A–A–B–B–A. After each hiding, the cat was allowed to find the food. According to the test group conditions, both A and B hiding events were accompanied by either ostensive, or non-ostensive verbal cues. We considered a choice as ‘perseveration’ if the subject chose location A when the bait was at location B.

Our main questions were: (i) does the presence or absence of ostensive verbal cues affect the choice pattern of cats; and (ii) does the familiarity of the human demonstrator (unknown experimenter vs. the owner of the cat) affect the choice behaviour of cats? Regarding the possible role of ostensive cues, we predicted that if we find no effect of these on the perseverative errors of cats, this would indicate that the companion animal status of cats does not necessarily require human- (or dog) analogous social understanding capacities. This could be explained by the fact that the domestic cat originated from a less gregarious ancestor than the hypothetical ancestor of the dog (Bradshaw 2016), and/or cats were either not selected for participating in cooperative tasks with humans, or usually do not receive the necessary amount of social experience to develop this skill. However, if our results would show a similar association in cats between the ostensive cues and perseverative errors than was found in dogs (and human infants), it would prove that independent of the level of sociability of the ancestral (pre-domestication) species, the selective pressure and environmental effects (i.e. learning) in the anthropogenic niche can result in analogous social skills in very different species as well (Wilkins et al. 2014).

Regarding our second question (the familiarity of the human partner—a factor that has been unfortunately not tested in case of dogs and socialized wolves earlier), we predicted that cats will have a higher rate of perseverative errors when their owner acts as demonstrator. The average companion cat’s acceptance level for strangers is lower than what we expect from dogs (Adamelli et al. 2005; Miklósi et al. 2005), which may predict a stronger response to the ostensive cues of the owner. Additionally, we also tested the possible effect of keeping conditions of the subjects (cats kept only outdoors; only indoors; outside/inside kept ones). There are indications that those companion animals that are kept exclusively outside of the house may get involved in less intense social interactions with their owners (McCabe and Ecker 1996; Clancy et al. 2003), therefore, we predicted that cats living solely outdoors would show less effect of human ostension. We also tested whether cats’ responses are associated with the presence or absence of dogs in the same household, because earlier results showed that dog owners may treat their cats differently, depending on the uni- or multipet household status (Pongrácz and Szapu 2018). Based on this, one could expect that dog owners get involved in less complex social interactions with their cats than sole cat owners, which in turn may be associated with a weaker reaction to human ostension in case of cats that share the environment with dogs. Finally, in a separate control experiment, we tested whether cats can find the hidden food based only on its scent, without any cues from the human demonstrator.

## Materials and methods

### Ethical note

Subjects of the experiments were privately owned companion cats. All procedures involving the cats and their owners were approved by the Ethical Committee of Eötvös Loránd University (Permission # PE/EA/1005-5/2018), and conducted in accordance with the recommendations of the Hungarian State Health and Medical Service. The owners took part with their cats in the test on a voluntary basis and they were informed that they would participate in a scientific study. Cat owners were present at the tests and they were told that they can terminate the experiment at any time when they think their cat is under unwanted stress.

### Subjects

We tested companion cats living with their owners (*N* = 38: 5 purebreds (2 Maine coons, 1 Scottish fold, 1 Persian, 1 British shorthair), 33 non-purebred cats. The sex ratio among the subjects was balanced 19 males (4 intact, 15 neutered), 19 females (7 intact, 12 spayed). The age of the subjects was between 6 months and 10 years. Participation was on a voluntary basis, the owners were recruited via online advertisement and personal acquaintance. We defined the following criteria for the possible participants: the cat should be between the age of 6 months and 10 years, it should live with its owner in the same household for at least 1 month before the experiment, it should listen to its name (i.e. it should come to the owner when he/she calls its name), it should be friendly with strangers and strongly motivated by food or a toy. To find out if the particular cat was an appropriate subject for the test, we did a three-phase preliminary test (Pongrácz et al. 2019). All subjects were naïve as they did not participate in any scientific test previously. Twenty-five subjects were tested in three contexts in a semi-random order: owner-ostensive context was always the third test, while we swapped the order of the experimenter ostensive and experimenter non-ostensive tests. As the owner non-ostensive context was included in the research after the completion of the 3 other contexts with 25 subjects, it was the last context tested. From the 25 subjects, 7 participated in all 4 contexts. Thirteen cats participated in the owner non-ostensive context alone.

### Preliminary test (based on Pongrácz et al. 2019)

The preliminary test consisted of three phases that followed each other in consecutive order. The experimenter (always the same 24-year-old woman, D.L.O.) performed the preliminary test when she visited the particular subject for the first time.

Phase 1: the experimenter called the subject by its name and other attention-calling sounds. The subject passed this phase if it approached the experimenter on its own without any coercion.

Phase 2: the experimenter approached the subject and attempted to pet it. The subject passed this phase if it allowed petting.

Phase 3: the experimenter put two small metal cups (we used two identical cups, depth 1 cm, diameter 4.5 cm) in front of the subject and placed a little piece of food in each of them. The food reward was chosen by the owners based on their previous experiences of their cats’ favourite treats. In case of each subject, some sort of semi-moist food item was used (e.g. various brands of cat treats, cheese, cold cuts, meat). The subject passed this phase if it ate the food from the metal cups. Based on the whole preliminary test, the subject was considered as suitable for the experiment if it passed at least two of the three phases. The third phase was also used to familiarize the subject with the knowledge that the cups may contain food. All subjects that eventually took part in the main test passed the third phase.

### General methods

After passing the preliminary test, all subjects moved immediately on to the main test. We did not do any kind of pre-training on the main experimental set-up.

The experiments were conducted in the subjects’ home to exclude a behaviour-changing effect of an unknown environment (see the same reason in Miklósi et al. 2005; Pongrácz et al. 2019). For cats that were kept exclusively indoors and those who had the opportunity to go outside, we chose a sufficiently large area in the house where there was enough open space to set up the camera and the hiding locations. For exclusively outdoor cats, we set up the test equipment in the yard of the house. The owners were asked not to feed the cats for a few hours before the start of the test. Therefore, experiments were scheduled according to the feeding time of the particular subject; or in the case of cats that were normally fed ad libitum, the food was withdrawn a couple hours before testing.

Before starting the main test, all subjects were given the opportunity to examine the test equipment (without the food bait placed) for several minutes. The experimenter set up the camera next to her left or right side facing towards the testing area diagonally, so both the subject, which was facing towards the experimenter, and the hiding locations, could be seen on the video footage. The two hiding screens (material: opaque plastic, 24 × 14 × 7.5 cm) were set 40 cm apart from each other and the metal cups were placed directly behind the screens. Each test was recorded with a Panasonic Lumix DMC-F2 camera.

### Main test (A-not-B error test)

Before starting the test, the owner was given instructions regarding what to do (and what not to do) during the test. The experimenter rubbed the chosen food bait onto the back of both the hiding screens and the inside of the metal cups to exclude scent-based searching as much as possible. The subject and the experimenter took up the starting position: the experimenter kneeled behind the hiding screens equidistant from each screen. The owner kept the subject at the starting point 1–1.5 m away from the screens, facing towards the experimenter and the screens. (In the case of the ‘owner ostensive’ condition, the role and position of the experimenter were swapped, otherwise everything was the same as we described it before). At the starting point, cats were kept in place by a gentle restraining by hand, meanwhile, the animal was sitting/standing on the floor. Twenty-five subjects were tested in each of 3 testing conditions (experimenter ostensive, experimenter non-ostensive, owner ostensive). We had minimum of 1 week to a maximum of 1 month break in between the testing occasions to minimize the effect of being trained for choosing correctly. The version of A-not-B error test, we employed here consists of five trials in a consecutive hiding order A–A–B–B–A (Péter et al. 2015). The five trials were recorded on video continuously. After the completion of the first three experimental conditions, an additional group was tested (owner non-ostensive). In this group, 7 subjects from the original 25, plus 13 additional cats were tested.

At the beginning of the trial, the experimenter held up the food bait in one hand in the middle, equidistant from each screen, and in ostensive context engaged the subject’s attention to herself by giving ostensive cues: calling the cat’s name, saying ‘Look!’ and using specific cat calling noises that are widely known and used (a sort of “tse” “tse” sound) until the subject made eye contact with the experimenter. Then, the experimenter moved the bait behind one of the screens in a straight line (and left the bait in the cup behind that particular screen), while still talking ostensively to the subject. Consequently, always only one cup had food bait in it. Then, she moved her hands behind her back. Then, the owner released the cat and let it choose one of the screens. If the subject chose correctly, it was allowed to eat the bait and then the owner gently returned it to the starting position. In the case of an incorrect choice, the subject was not allowed to have the food (the person who performed the hiding of the food, picked up the ‘unchosen’ cup before the subject could visit it). After the subject made its choice, both cups were removed shortly, to prevent the cat from visiting the other cup. The hiding method was the same in the three other contexts. In the experimenter non-ostensive and the owner non-ostensive contexts, the experimenter and the owner recited a short poem in a non-ostensive manner during the hiding process. In the owner-ostensive context, the owner called the cat by name and hid the bait, performing identically to the experimenter ostensive context.

All pieces of the equipment were sanitized with antibacterial gel before and after every test (except between cats living in the same household).

### Exclusion of subjects and temporary suspending of the test

Only cats who passed at least two phases of the preliminary test were allowed to participate in the main test. Here, in the event that something distracted the subject’s attention during any of the test trials and it did not start searching within 20 s after being released, the trial was repeated. If the cat did not choose any of the screens after the third repeat of a given trial, the experiment was postponed and repeated from the beginning a minimum of 1 week later. If the subject still did not cooperate, after another week, the experimenter returned one more time. If the cat could not complete the test upon the third occasion, it was excluded from the experiment. Furthermore, subjects were excluded from the final group of participants due to their age (they were older than 10 years), or had no willingness to cooperate with the experimenter and their owner (they did not show interest towards the task, and or showed aggressive behaviour if handled).

The experimenter visited 68 possible subjects. From these, 2 cats were excluded due to their age, 7 failed during the preliminary test, and 21 refused further cooperation after some trials. From the remaining 38 cats, 7 participated in and finished all 4 contexts, 18 participated in only 3 (experimenter ostensive, experimenter non-ostensive and owner ostensive) contexts and 13 cats only participated in the owner non-ostensive context.

### Data extraction from the videos

The video footages were analysed by the experimenter. Success of choice was determined by whether or not the subject chose the baited screen. If it did, the trial was considered successful, if it did not, the trial was unsuccessful. The choice itself was defined as ‘made’ when the subject obviously looked behind one of the screens and approached the metal cup behind a screen closer than 10 cm.

From the total 475 trials, an independent coder re-analysed 135, and the inter-coder agreement was found very high (Cohen’s Kappa = 0.941; *P* < 0.001).

### Scent-based searching—control experiment

For ruling out the hypothesis that subjects chose the baited screen based on their olfactory sense (due to the asymmetrically located food bait), we ran a short control test with a group of cats, which were chosen randomly from the participants eligible for the main tests. We used the same hiding screens and metal cups as in the A-not-B error test. The screens and the cups were previously rubbed with the food bait. The hiding event was repeated ten times. The food was hidden behind the two screens in a semi-random order (the first and second hiding could not be behind the same screen and the food was not hidden behind the same screen more than two times in a row).

For the test, the experimenter and the subject took the starting position. To prevent additional (non-olfactory) cueing, neither the experimenter, nor the owner, gave any sounds during the hiding event or the search. The experimenter put the metal cups in front of her in the middle, on top of each other. Then, she conspicuously placed a piece of food in the cup on the top. She lifted both cups in middle line at the same time, then simultaneously placed the cups behind the screens. From the cat’s point of view, the food bait was invisible in the cup, because the rim of the cup was higher than the diameter of the piece of food. After she put her hands behind her back, the subject was released to search for the food. If the cat chose the baited screen, it was allowed to eat the reward, but in the case of an incorrect choice, the bait was removed from the cup and the cat was returned to the starting position by the owner for the next trial. If the cat did not choose (withdrew itself from the testing context by leaving the testing area, and or performing replacement activities such as cleaning itself), the owner took the cat back to the starting position and the trial was repeated a maximum of three times. If the subject did not choose by the third repeat, the experimenter terminated the test. Only the results of tests with ten completed trials (regardless of the choices made) were included in the final statistics. From the initial 14 subjects, 10 cats made at least 10 valid choices in the scent control experiment.

### Data analysis

All the analyses were performed with IBM SPSS (22). In each trial, we used Binomial tests to determine whether cats’ correct choices differed significantly from chance level (set at 0.5). To account for the increased chance of type I errors due to multiple comparisons, we adjusted the *P *values in each testing condition using the method by Hochberg (1988). Therefore, *P* values in case of the Binomial tests are marked as *p*_hoch_, indicating that they have been adjusted accordingly. In each analysis, *α* = 0.05 was used.

Within each testing condition, we analysed with Cohran *Q*-tests whether cats chose with different success rate in the individual trials. In the case of a significant main effect, we used McNemar paired comparisons to indicate significant between-trial differences.

With Cohran *Q*-tests and McNemar paired comparisons, we analysed if there was a difference among the success rates of cats across each testing conditions’ B3 trials.

Finally, we also examined whether the housing conditions or the presence of a dog in the same household had an effect on the success rate in B3 and B4 trials. We used Kruskal–Wallis test for housing conditions and Mann–Whitney U test for the dog’s presence.

## Results

### Scent control experiment

On the individual level, only one cat from the ten found the food reward with a success rate significantly above chance (nine out of ten times). On the group level, cats’ success rate was at chance level (one-sample *t* test, *t*(9) = 0.171; *P* = 0.868). Therefore, we can conclude that the success rate of the cats was not likely to be influenced by olfactory cues in the main tests either.

### Main tests: success rates in the individual trial categories

The success rate of cats was significantly above chance level in most trials (Binomial test), with the exception of trials B3 in each except for the ostensive experimenter condition and trial B4 in the non-ostensive experimenter condition (Table S1). Cats chose at chance level in trials B3 (non-ostensive experimenter, ostensive and non-ostensive owner) and also in trial B4 (non-ostensive experimenter).

### Within-condition comparisons of the success rates of trials

**Fig. 1:**
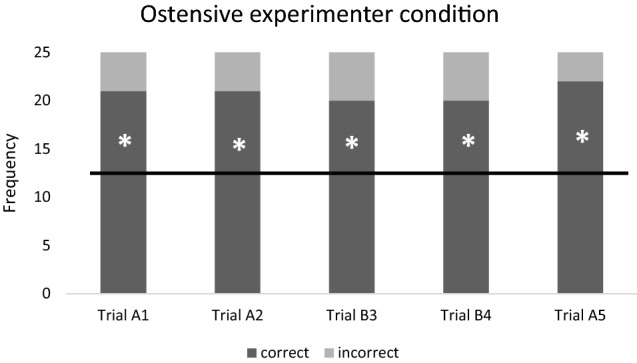
Ratio of the correct and incorrect choices in the ‘Ostensive experimenter’ condition. Horizontal line marks the chance level. *N* = 25 subjects. * = success rate is significantly different from the chance level

**Fig. 2:**
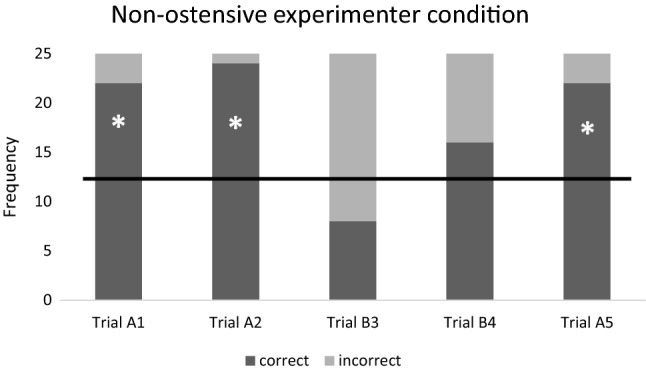
Ratio of the correct and incorrect choices in the ‘Non-ostensive experimenter’ condition. Horizontal line marks the chance level. *N* = 25 subjects. * = success rate is significantly different from the chance level

**Fig. 3:**
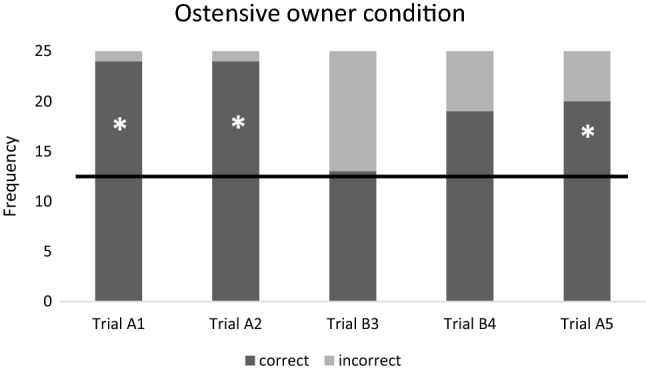
Ratio of the correct and incorrect choices in the ‘Ostensive owner’ condition. Horizontal line marks the chance level. *N* = 25 subjects. * = success rate is significantly different from the chance level

**Fig. 4:**
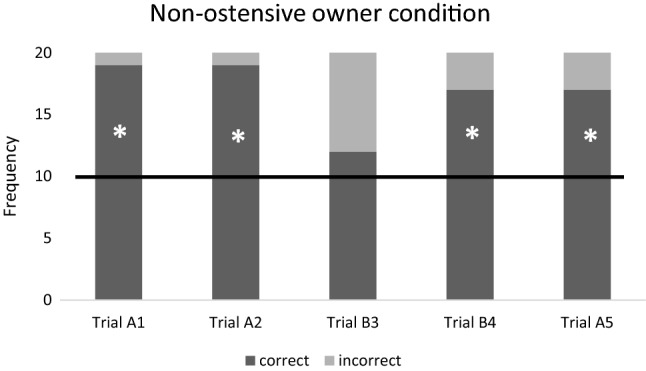
Ratio of the correct and incorrect choices in the ‘Non-ostensive owner’ condition. Horizontal line marks the chance level. *N* = 20 subjects. * = success rate is significantly different from the chance level

In the ostensive experimenter condition, we did not find significant difference among the individual trials’ success rates (repeated Cohran *Q* test, *N* = 25; *Q*_4_ = 0.824; *P* = 0.935, Fig. 1). In the non-ostensive experimenter condition, success rate showed a significant trial effect (*N* = 25; *Q*_4_ = 32.240; *P* < 0.001), where cats chose with a significantly lower success rate in trial B3 than in any of the other trials (Fig. 2). Cats chose with significantly different success rates also in the ostensive owner condition (*N* = 25; *Q*_4_ = 21.579; *P* < 0.001). According to the post hoc tests, the success rate of cats was significantly lower in trial B3 than in trials A1, A2 and A5 (Fig. 3). Finally, in the non-ostensive owner condition, again we found a significant main effect (*N* = 20; *Q*_4_ = 11.310; *P* = 0.023). According to the post hoc tests, cats’ success rate was significantly lower in trial B3 than in trials A1 and A2 (Fig. 4).

### Between-condition comparison of B3 success rates

We found a significant main effect of testing condition when compared the B3 trials among the ostensive experimenter, non-ostensive experimenter and ostensive owner groups (repeated Cohran *Q* test, *N* = 25; *Q*_4_ = 12.111; *P* = 0.002). According to the McNemar post hoc comparisons, cats had significantly lower success rate in the B3 trial of the non-ostensive experimenter than in the ostensive experimenter group. Success rates did not differ significantly between any of the other pair-wise comparisons of conditions.

### Associations of cats’ success rate in B3 and B4 trials with keeping location and the presence of dogs in the household

We found no significant associations between the keeping location and success rates in the B3 and B4 trials. Similarly, having a dog or not in the same household did not have an association with the performance of cats (Table S2).

## Discussion

In our study, the main findings were the following: domestic cats could successfully find the hidden food in each of the five trials only when the experimenter did the hiding with simultaneous ostensive verbal cueing. In all the other experimental conditions (non-ostensive experimenter; ostensive and non-ostensive owner), cats’ performance dropped to the chance level in the B3 trial, which indicates that apart from the ostensive experimenter group, a considerable proportion of cats committed perseverative (A-not-B) errors in the B3 trial. We also confirmed with a scent control experiment, that the performance of the cats in the two-way choice paradigm is unlikely to be driven by quantitative differences in scent cues between the baited and non-baited locations.

According to our original hypotheses, familiarity with the human partner and the specific sensitivity to human ostensive cues would enhance the frequency of perseverative errors committed by cats in the B-trials. One could expect that cats would show higher rate of perseverative errors when (i) their owner did the hiding of the food and (ii) he/she used ostensive cueing during the hiding act. However, if cats would be unaffected by ostensive cues, one could expect an even (low or zero) rate of perseverative errors across and within the experimental groups. Our results showed the effect of both the familiarity of the human demonstrator and the type of verbal cueing; however, we found a more or less different pattern of associations than expected. Table S3 provides an easy-to-understand comparison between our findings on cats and the results of wolves, dogs and human toddlers (Topál et al. 2009).

Cats performed with a steadily high success rate (i.e. no perseveration in trial B3) in the ostensive experimenter condition. If we add to this, the results of the ostensive owner group, where cats more often committed perseverative errors in the B3 trial, one could assume that familiarity with the human partner enhances the attention and subsequently a fast ‘rule-learning’ response of cats, eventually resulting in a similar pattern of performance as was found earlier in dogs and human infants (Topál et al. 2009; Péter et al. 2015). In this case, the difference among dogs and cats would reveal itself in the fact that meanwhile dogs committed perseverative errors when an unfamiliar experimenter performed the object hiding with simultaneous ostensive cueing, in cats only the most familiar person (the owner) could elicit this effect. In the case of domestic cats, their rather rigid territorial lifestyle may not have generated an ecological need for a fast acceptance of strangers (Miklósi et al. 2005; Pongrácz et al. 2019). Furthermore, an average companion dog is almost surely exposed to strangers than the average companion cat is—which again could bias the responsiveness of cats toward the familiar owner’s cues.

However, Vitale and Udell (2019) showed that companion cats are ready to establish social contact with unfamiliar humans just as likely as with their owner. In agreement with this, our results of the non-ostensive experimenter and non-ostensive owner conditions also do not support the theory outlined in the previous paragraph. In these groups, the success rate of cats was at chance level in the B3 trials—a similar result that we found in the ostensive owner group. Some authors concluded that a high tendency for perseveration in B3 trials was due to the subjects’ (human infants/ dogs) specific sensitivity to ostensive cueing, and if the subjects did not commit the A-not-B error, it was either the sign that they are not sensitive to human ostensive cues (in socialized wolves—Topál et al. 2009), or because there were no ostensive cues (human infants/ dogs—Topál et al. 2008; Péter et al. 2015). It seems that in the case of cats, the frequency of perseveration raises in each condition apart from the ostensive experimenter group. This indicates that cats’ reliance on ostensive cues is most probably based on different socio-cognitive mechanisms or learning history than those of dogs or human infants. In dogs, according to the theory of Topál et al. (2009), ostensive signals elicit an effective and rapid learning of human-demonstrated ‘rules’, such as the preference for a firstly reinforced search location (location ‘A’)—and this leads to the perseverative error during the subsequent B-trials. According to our results, cats persevere both in ostensive and non-ostensive trials, and they do it also somewhat independently of the familiarity of the human demonstrator. Their steadily high success rate in the ostensive experimenter group shows however, that one particular combination of the demonstrator-ostension conditions resulted in an almost complete disappearance of perseveration in the B3 trial. One way to explain this would be that in cats, perseveration occurs when the cues (verbal plus motoric) provided by the demonstrator are either confusing (non-ostensive owner) or not strong enough (non-ostensive experimenter) for engaging the cats’ attention. In this case, cats may revert to a win-stay search strategy (Warren 1966; Levine et al. 1987), which enhances their preference for location A, causing a temporary peak of perseveration in trial B3. As cats have been found to have only a limited duration of memory for disappearing objects (Fiset and Doré 2006), any disturbance in their attention may cause a shift to the previously rewarded location. In this framework, where perseveration would be caused in cats by a temporarily less intense attention, our subjects’ almost perfect performance in the ostensive experimenter group could be explained by the superposition of two attention-eliciting factors: the novelty effect of the unknown experimenter and the ostensive signals. Thus, unlike in dogs, ostension (by a friendly stranger) would result in an almost complete absence of perseveration. This result would be indirectly supported by our earlier finding, where in a gaze-following task, ostensive verbal signals of an unknown experimenter resulted in faster establishment of eye contact with cats (Pongrácz et al. 2019).

As a limitation of our study, we have to mention that beyond the assumed difference between their ancestor species’ social predisposition and the differences between the aspects of their after-domestication selection history, individual companion cats and dogs are most likely exposed to very different social experiences throughout their lifetime. An average dog is exposed to much more various and numerous encounters with unfamiliar people than a companion cat. Similarly, owners are engaged in more training and task-like interactions with their dogs than with their cats. Therefore, the A-not-B test in itself could be a less stressful and strange event for the dog subjects of the earlier papers (Topál et al. 2009; Péter et al. 2015) than for the cats in our experiments. At the same time, we may remind the reader that with our preliminary test, we sorted out those cats that were totally unable/unwilling to cooperate with the unfamiliar experimenter—a detail that usually remains obscure in case of dog (or human infant) subjects of a wide array of studies in comparative cognition.

As a conclusion, cats not only show the capacity for highly developed social skills regarding reliance on various human-given signals (e.g. pointing—Miklósi et al. 2005; gazing—Pongrácz et al. 2019), and to form attachment with their owner (Vitale et al. 2019), their responses to human attention-eliciting behaviour also shows markedly unique features. Behavioural scientists should be aware that the many times similar performance in cats, compared to dogs in socio-cognitive tasks, may be the result of different mechanisms—as in our case, perseveration in the two-way object hiding task in cats is more likely the result of reverting to the win-stay strategy, than a strong reliance on the ostensively highlighted choice shown by a human demonstrator.

## Electronic supplementary material

Below is the link to the electronic supplementary material.

Supplementary file1 (DOCX 17 kb)

Supplementary file2 (XLSX 25 kb)
