# Supplementary material for: Cats show an unexpected pattern of response to human ostensive cues in a series of A-not-B error tests
Source: Anim Cogn. 2020 Mar 29;23(4):681–9. doi: 10.1007/s10071-020-01373-4 (PMC7320938; doi:10.1007/s10071-020-01373-4)
Supplement: Supplementary file 1 — Supplementary file1 (DOCX 17 kb) [file 10071_2020_1373_MOESM1_ESM.docx]

Table S1

| Condition | Trial | N | *p* | Rank | *p*_hoch_ |
| --- | --- | --- | --- | --- | --- |
| Ostensive experimenter | **A1** | **25** | **<0.0001** | **3** | **0.03** |
|  | **A2** | **25** | **<0.0001** | **3** | **0.03** |
|  | **B3** | **25** | **0.0041** | **7** | **0.07** |
|  | **B4** | **25** | **0.0041** | **7** | **0.07** |
|  | **A5** | **25** | **<0.0001** | **4** | **0.04** |
| Non-ostensive experimenter | **A1** | **25** | **<0.0001** | **4** | **0.04** |
|  | **A2** | **25** | **<0.0001** | **1** | **0.01** |
|  | B3 | 25 | 0.1081 | 9 | 0.09 |
|  | B4 | 25 | 0.2301 | 10 | 0.10 |
|  | **A5** | **25** | **0.0001** | **5** | **0.05** |
| Ostensive owner | **A1** | **25** | **0.0001** | **5** | **0.05** |
|  | **A2** | **25** | **0.0001** | **5** | **0.05** |
|  | B3 | 25 | 1.0001 | 11 | 0.12 |
|  | B4 | 25 | 0.0151 | 8 | 0.08 |
|  | **A5** | **25** | **0.0041** | **7** | **0.07** |
| Non-ostensive owner | **A1** | **20** | **<0.0001** | **2** | **0.02** |
|  | **A2** | **20** | **<0.0001** | **2** | **0.02** |
|  | B3 | 20 | 0.5031 | 12 | 0.11 |
|  | **B4** | **20** | **0.0031** | **6** | **0.06** |
|  | **A5** | **20** | **0.0031** | **6** | **0.06** |

Comparison of cats’ performance to the chance level (0.5) across the test trials in each condition (Binomial test). P-values are adjusted according to the Benjamini-Hochberg method for controlling the false discovery rate (Type I errors). *p*_hoch_ was calculated by following the equation *(i/m)Q*, where *i* is the rank, *m* is the total number of tests, and *Q* is the chosen false discovery rate (0.20 in this case). After-adjustment significant results are highlighted with bold typesetting.

**Table S2**

| **Factor** | **Condition** | **trial B3** | **trial B4** |
| --- | --- | --- | --- |
| keeping location | ostensive experimenter | κ^2^(2)=2.400; P=0.301 | κ^2^(2)=1.007; P=0.604 |
|  | non-ostensive experimenter | κ^2^(2)=1.106; P=0.575 | κ^2^(2)=1.358; P=0.507 |
|  | ostensive owner | κ^2^(2)=2.407; P=0.300 | κ^2^(2)=0.165; P=0.921 |
|  | non-ostensive owner | κ^2^(1)=0.000; P=1.000 | κ^2^(1)=0.124; P=0.725 |
| dog in household | ostensive experimenter | U=45.500; P=0.127 | U=58.000; P=0.663 |
|  | non-ostensive experimenter | U=60.000; P=0.822 | U=60.000; P=0.822 |
|  | ostensive owner | U=46.000; P=0.235 | U=59.000; P=0.744 |
|  | non-ostensive owner | U=17.000; P=0.884 | U=13.000; P=0.402 |

Results of the Kruskal-Wallis tests (keeping condition) and Mann-Whitney U-tests (dog in the household). Neither of these independent factors had any significant association with the cats’ success rate in trials B3 and B4 (α=0.05).

**Table S3**

|  |  | **Perseveration** | | | |
| --- | --- | --- | --- | --- | --- |
| **Demonstrator** | **Signaling** | **Wolves^1^** | **Infants^1^** | **Dogs^1^** | **Cats^2^** |
| **Unfamiliar** | non-ostensive | No | No | No | Yes |
|  | ostensive | No | Yes | Yes * | No |
| **Familiar** | non-ostensive | - | x | x | Yes |
|  | ostensive | - | x | x | Yes |

A summary of the results of comparably executed A-not-B error tests with wolves, human infants, dogs and cats as subjects. Note, that only the cats were tested also with a familiar person (owner) as demonstrator.

^1^ results are taken from Topál et al. (2009)

^2^ results are reported in the current paper

* Dogs did not commit perseverative errors, if the human who performed the (ostensive) A trials was not the same as the person who performed the B trials.
